# Erdafitinib in Asian patients with advanced solid tumors: an open-label, single-arm, phase IIa trial

**DOI:** 10.1186/s12885-024-12584-0

**Published:** 2024-08-13

**Authors:** Joon Oh Park, Yin-Hsun Feng, Wu-Chou Su, Do-Youn Oh, Bhumsuk Keam, Lin Shen, Sang-We Kim, Xiufeng Liu, Huimin Liao, Min Qing, Chong Zhang, Jiaqi Qian, Xiaodan Tang, Peng Li, Spyros Triantos, Hussein Sweiti

**Affiliations:** 1 Division of Hematology-Oncology, Department of Medicine, Samsung Medical Center Sungkyunkwan University School of Medicine, Seoul, Korea; 2 Division of Hematology and Oncology, Department of Internal Medicine, Chi-Mei Medical Center, Tainan, Taiwan; 3 Department of Oncology, National Cheng Kung University Hospital, Tainan, Taiwan; 4 Department of Internal Medicine, Seoul National University Hospital, Seoul, Korea; 5 Department of Internal Medicine, Cancer Research Institute, Seoul National University Hospital, Seoul National University College of Medicine, Seoul, Korea; 6 Department of GI Oncology, Peking University Cancer Hospital & Institute, Beijing, China; 7 Oncology, Asan Medical Center, University of Ulsan College of Medicine, Seoul, Korea; 8 Qinhuai Medical Zone, Eastern Theater General Hospital of the Chinese PLA, Nanjing, China; 9 Janssen China R&D Center, Shanghai, China; 10 Oncology Clinical Development, Janssen R&D, PA USA

**Keywords:** Cholangiocarcinoma, NSCLC, Erdafitinib, FGFR inhibitor, Asian patients

## Abstract

**Background:**

*FGFR* genomic aberrations occur in approximately 5–10% of human cancers. Erdafitinib has previously demonstrated efficacy and safety in *FGFR*-altered advanced solid tumors, such as gliomas, thoracic, gastrointestinal, gynecological, and other rare cancers. However, its efficacy and safety in Asian patients remain largely unknown. We conducted a multicenter, open-label, single-arm phase IIa study of erdafitinib to evaluate its efficacy in Asian patients with *FGFR*-altered advanced cholangiocarcinoma, non-small cell lung cancer (NSCLC), and esophageal cancer.

**Methods:**

Patients with pathologically/cytologically confirmed, advanced, or refractory tumors who met molecular and study eligibility criteria received oral erdafitinib 8 mg once daily with an option for pharmacodynamically guided up-titration to 9 mg on a 28-day cycle, except for four NSCLC patients who received erdafitinib 10 mg (7 days on/7 days off) as they were recruited before the protocol amendment. The primary endpoint was investigator-assessed objective response rate per RECIST v1.1. Secondary endpoints included progression-free survival, duration of response, disease control rate, overall survival, safety, and pharmacokinetics.

**Results:**

Thirty-five patients (cholangiocarcinoma: 22; NSCLC: 12; esophageal cancer: 1) were enrolled. At data cutoff (November 19, 2021), the objective response rate for patients with cholangiocarcinoma was 40.9% (95% CI, 20.7–63.6); the median progression-free survival was 5.6 months (95% CI, 3.6–12.7) and median overall survival was 40.2 months (95% CI, 12.4–not estimable). No patient with *RET/FGFR*-altered NSCLC achieved objective response and the disease control rate was 25.0% (95% CI, 5.5–57.2%), with three patients with stable disease. The single patient with esophageal cancer achieved partial response. All patients experienced treatment-emergent adverse events, and grade ≥ 3 treatment-emergent adverse events were reported in 22 (62.9%) patients. Hyperphosphatemia was the most frequently reported treatment-emergent adverse event (all-grade, 85.7%).

**Conclusions:**

Erdafitinib demonstrated efficacy in a population of Asian patients in selected advanced solid tumors, particularly in those with advanced *FGFR*-altered cholangiocarcinoma. Treatment was tolerable with no new safety signals.

**Trial registration:**

This trial is registered with ClinicalTrials.gov (NCT02699606); study registration (first posted): 04/03/2016.

**Supplementary Information:**

The online version contains supplementary material available at 10.1186/s12885-024-12584-0.

## Introduction

Fibroblast growth factor receptors (FGFRs) are a family of receptor tyrosine kinases involved in the regulation of cellular function affecting cell growth, development, differentiation, and survival, among others [1]. The *FGFR* family consists of four highly conserved transmembrane receptors (*FGFR1–4*) and a related receptor, *FGFR5,* which lacks an intracellular kinase domain [1, 2]. Aberrations in the *FGFR1–4* gene resulting from gene fusions, rearrangements, somatic mutation, or amplification have been reported in many forms of malignancies [3]. Reported prevalence rates for all human cancers that harbor *FGFR* aberrations have typically been 5–10%, but this frequency is higher most notably in urothelial cancer and cholangiocarcinoma at 10–30% [1], and 10–15% in intrahepatic cholangiocarcinoma [4]. A recent cross-sectional study in China identified *FGFR* aberrations (including *FGFR* single-nucleotide variants, fusion, and amplification) in 7.0% of cancer patients, occurring at varying frequencies across different tumor types, with prevalence ranging from 30.5% in urothelial cancer, 16.9% in endometrium cancer, 14.5% in esophageal cancer, and 13.2% in breast cancer to 8.8% in lung cancer [5]. In another large study of 10,966 patients with non-small cell lung cancer (NSCLC), *FGFR* mutations were reported in 1.9% of the population [6].

Cholangiocarcinoma is the second most common primary liver cancer after hepatocellular carcinoma, comprising around 10–15% of all liver tumors [7]. Mortality rate of cholangiocarcinoma appears to be higher in Asia than in Europe or North America [8]. Therapeutic options are limited for patients with advanced disease, especially in the second-line setting, and the outcomes for palliative chemotherapy are poor [9]. Early efforts to target *FGFR* in cholangiocarcinoma were non-selective and were met with less favorable off-target side effects [4]. Selective FGFR inhibitors against cholangiocarcinoma have since been developed and showed promising results in the advanced metastatic setting, with responses ranging from 20 to 40% [4, 10–13]. Lung cancer, on the other hand, is the most common cause of cancer-related death worldwide [7]. Immune checkpoint inhibitors with or without chemotherapy are the preferred first-line treatment in advanced programmed death-ligand 1–positive NSCLC without oncogenic driver mutations [14]. However, a significant proportion of patients still experience disease progression [15]. *FGFR* alterations or amplifications are reported in patients with NSCLC. Moreover, *FGFR* mutations may also develop as an acquired resistance mechanism for epidermal growth factor receptor (EGFR) tyrosine kinase inhibitors (TKIs). Preliminary treatment outcomes with FGFR TKIs in NSCLC have been modest with infigratinib reporting an overall response of 11% among 48 patients and a low objective response rate (ORR) of 5% with rogaratinib among 20 patients in phase I studies [16, 17]. More recent data with erdafitinib showed an ORR of 26.1% in 23 patients with NSCLC and select *FGFR* mutations or fusions [18]. Esophageal cancer is an aggressive disease which ranks seventh and sixth in terms of incidence and mortality worldwide, respectively [7]. Eastern Asia has the highest incidence of esophageal cancer, with a large burden in China [7]. Although there are several novel targeted therapies for esophageal cancer, radiation therapy and chemotherapy are common, and the outcomes with these treatments are poor in advanced esophageal cancer [19]. As such, these three solid tumors account for a large burden in many parts of the world, especially in Asia. In this regard, FGFR inhibitors have emerged as an approach given the presence of *FGFR* aberrations in these tumor types. FGFR2b antibody showed promising results in patients with *FGFR2b* overexpressed gastric and gastro-esophageal junction adenocarcinomas [20].

To date, several selective FGFR inhibitors are under clinical investigation in a variety of *FGFR*-altered tumor types. Four of them have been approved by the U.S. Food and Drug Administration for the treatment of urothelial cancer or cholangiocarcinoma, including erdafitinib, pemigatinib, futibatinib, and infigratinib [21–24]. Erdafitinib was the first selective oral pan-FGFR inhibitor approved in 2019 for the treatment of locally advanced or metastatic urothelial carcinoma with susceptible *FGFR2/3* alterations that has progressed during or following at least one line of platinum-containing chemotherapy, including within 12 months of neoadjuvant or adjuvant platinum-containing chemotherapy [21, 25]; long-term follow-up confirmed its efficacy and safety [26]. Erdafitinib has also demonstrated tumor-agnostic efficacy and safety in *FGFR*-altered advanced solid tumors, including gliomas, thoracic, gastrointestinal, gynecological, and rare cancers [27]. More recently, in a phase III study, erdafitinib showed longer overall survival than chemotherapy among patients with advanced or metastatic urothelial carcinoma after previous immunotherapy [28]. However, less is known about its effectiveness in Asian patients for various tumor types. The present phase IIa study was therefore conducted to investigate the antitumor activity of erdafitinib in Asian patients with *FGFR*-altered advanced solid tumors.

## Patients and methods

### Patients

Eligible participants were adult patients (aged ≥ 18 years) with pathologically or cytologically confirmed advanced or refractory tumors (pre-defined in the protocol to enroll squamous and non-squamous NSCLC, esophageal cancer, urothelial cancer, and cholangiocarcinoma) who met molecular eligibility criteria (i.e., all *FGFR* gene translocations, *FGFR* gene mutations, or with evidence of *FGFR* pathway activation or other potential/emerging targets/pathways) determined by a central or local laboratory using a tumor tissue-based assay. In addition, RET proto-oncogene (*RET*)-activating mutations or *RET* translocations were study-eligible for patients with NSCLC. Other key inclusion criteria included the presence of measurable disease (per Response Evaluation Criteria in Solid Tumors version 1.1 [RECIST v1.1]) and documented disease progression as defined by RECIST v1.1 at baseline, [11] and an Eastern Cooperative Oncology Group performance status (ECOG PS) of 0 or 1. Patients were required to have adequate bone marrow, liver, and kidney function. A full account of the study eligibility criteria, including details of the molecular eligibility criteria, is described in Supplementary Table S1.

### Study design and treatment administration

This was an open-label, multicenter, single-arm phase IIa study to evaluate the clinical efficacy, safety, and pharmacokinetics of erdafitinib in Asian patients with advanced NSCLC, urothelial cancer, esophageal cancer, and cholangiocarcinoma. The study was conducted at 10 study sites in China, South Korea, and Taiwan (ClinicalTrials.gov NCT02699606). Randomization and blinding were not applicable. Enrolled patients received oral erdafitinib 8 mg once daily (QD) with an option to up-titrate to 9 mg on a 28-day cycle after a protocol amendment (September 18, 2016) was implemented. Erdafitinib dose was up-titrated or maintained taking into account the phosphate levels measured on day 14 of cycle 1 and the toxicity observed to that day. Patients who started on erdafitinib 10 mg for 7 days on/7 days off (days 1–7 on, days 8–14 off, days 15–21 on, days 22–28 off), with an option of up-titration based on serum phosphate levels measured on cycle 1 day 21 or observed toxicity before the amendment was implemented, continued with this treatment regimen. The reason to amend the protocol to adopt the continuous dosing regimen from the intermittent one was based on the observed data from the pharmacokinetic and pharmacodynamic modeling of serum phosphate level in a phase II study on urothelial carcinoma [25]. The continuous 8 mg dosing regimen was also more efficacious than the intermittent one and the adverse events were manageable with dose titration. Patients were allowed to receive treatment until the occurrence of disease progression, unacceptable toxicity, or any other protocol-defined reason for treatment discontinuation. The treating physician could continue with the study treatment in the best interest of the patient despite progressive disease until treatment was no longer considered beneficial.

The study was conducted in accordance with the principles of the Declaration of Helsinki and Good Clinical Practice guidelines and had the approval of local laws and regulations where the study was being conducted. All patients provided written informed consent according to local requirements to participate prior to screening. The trial protocol and its amendments were reviewed and approved by an independent ethics committee at each participating site.

### Outcomes and assessments

The primary endpoint was investigator-assessed ORR per RECIST v1.1. Secondary endpoints were progression-free survival (PFS), duration of response (DOR), disease control rate (DCR), overall survival (OS), safety, and pharmacokinetics.

Baseline radiological assessment was undertaken within 4 weeks before administration of the first dose of study drug. During the study, assessment of tumor responses was performed according to RECIST v1.1 by investigators using computed tomography (CT) scans of the locations of known disease. Magnetic resonance imaging was permitted to evaluate sites of disease that could not be adequately imaged using a CT scan. Disease evaluation was performed every 8 (± 1) weeks for a period up to 1 year after the start of study drug, and then every 12 weeks. Patients who discontinued the study drug before disease progression were evaluated every 8 (± 1) weeks until disease progression was documented, subsequent therapy started, death, or withdrawal of consent, whichever occurred first.

In the follow-up period, patients were assessed for survival every 8 (± 1) weeks and end-of-treatment for a period of up to a year after the start of study drug, then every 12 (± 1) weeks thereafter until death, withdrawal of consent, loss of follow-up, or conclusion of the study, whichever occurred first.

Safety was assessed based on the occurrence of adverse events (AEs), vital signs, electrocardiograms, echocardiograms, physical examinations, clinical laboratory tests, and ECOG PS at specified time points. All AEs were coded using MedDRA version 23.0, and severity was graded according to the National Cancer Institute Common Terminology Criteria for Adverse Events Version 4.0 (NCI-CTCAE v4.0). Hyperphosphatemia and AEs related to nails were graded per protocol (see Supplementary Table S2). Complete eye examination and Amsler grid testing were performed at screening, the beginning of every new cycle, and the end of treatment. Patients with abnormal Amsler grid testing were referred for full ophthalmologic examination within 7 days.

Serum blood samples were collected pre- and post-dose (3, 6, 24 h) on cycle 1 day 1 and 14, cycle 2 day 1, and pre-dose for cycle 3 and 4 day 1 for those who were on the 8 mg QD regimen. Those on 10 mg 7 days on/7 days off had their blood samples collected pre- and post-dose (3, 6, 24 h) on cycle 1 day 1 and 7 and cycle 2 day 1. Collected plasma samples were analyzed for erdafitinib concentration using a liquid chromatography with tandem mass spectrometry assay. An additional 4 mL blood sample was collected for the determination of the fraction unbound and protein levels (total protein, albumin, and alpha-1-acid glycoprotein [AGP]) on cycle 1 day 1 at 3 h post-dose.

### Statistical analysis

Sample size considerations assumed a hypothesized ORR of 35% for erdafitinib 8 mg QD versus a null hypothesis of 15% or less. Based on a one-sided type I error of 0.2, the study would be expected to have 72% power to reject the null hypothesis, with 13 patients with evaluable response. Hence, the enrollment of approximately 15 patients was planned under 8 mg QD for each planned histology subtype (squamous and non-squamous NSCLC) in Cohort A and each planned tumor type in Cohort B (urothelial cancer, esophageal cancer, and cholangiocarcinoma).

The efficacy analyses were primarily based on the treated population which consisted of all patients who received at least one dose of the study drug. Summaries of AEs and other safety data were based on 35 patients who received at least one dose of study drug.

ORR, DCR, and corresponding exact 95% confidence intervals (CIs) were calculated using the Clopper–Pearson method. Median and 95% CI values were estimated for DOR, PFS, and OS using the Kaplan–Meier method. All statistical analyses were performed using SAS version 9.4 software (SAS Institute, Inc, Cary, NC). This trial is registered with ClinicalTrials.gov (NCT02699606).

## Results

### Patients

Between August 22, 2016, and November 19, 2021, 344 patients were centrally screened for *FGFR*-activating mutations/rearrangement (urothelial cancer, esophageal cancer, NSCLC, and cholangiocarcinoma) or *RET*-activating mutations/translocations (NSCLC); 35 were enrolled across three sites in China, three sites in South Korea, and four sites in Taiwan (Supplementary Figure S1). The study enrolled mostly patients with cholangiocarcinoma and NSCLC, except for one patient with esophageal cancer (a 56-year-old male patient whose initial diagnosis was esophageal carcinoma with metastatic disease in the lung) who entered this trial before the enrollment scope changed. At the data cutoff (November 19, 2021) for final analysis, there were 22 treated patients with cholangiocarcinoma (10 [45.5%] from Taiwan, six [27.3%] from mainland China, and six [27.3%] from Korea), 12 patients with NSCLC (eight [66.7%] from Korea and four [33.3%] from Taiwan), and one patient with esophageal cancer from Korea. Of the 22 patients with cholangiocarcinoma, 16 (72.7%) were intrahepatic and five (22.7%) were extrahepatic. One (4.5%) patient had combined hepatocellular cholangiocarcinoma.

Patient demographics and baseline characteristics are summarized in Table 1. All patients with cholangiocarcinoma, eight patients with NSCLC, and one patient with esophageal cancer received erdafitinib 8 mg QD; four patients with NSCLC received 10 mg (7 days on/7 days off). At the cutoff date, 10 patients with cholangiocarcinoma completed the study; six were still on study, four were in survival follow-up, and two were still receiving treatment. Twenty patients with cholangiocarcinoma discontinued treatment, 19 were due to progressive disease and one due to death. All patients with NSCLC discontinued treatment, nine due to progressive disease, two due to AEs, and one withdrew. The patient with esophageal cancer had *FGFR3-TACC3* rearrangement and had received prior radiotherapy, prior cancer-related surgery, and systemic therapy. He discontinued treatment due to progressive disease. The median age of the overall patient population was 54 years (range, 25–78); 22 (62.9%) patients were male and 19 (54.3%) had an ECOG PS of 1. All patients had stage IV disease and had received at least one prior systemic therapy (Table 1). Tumors harboring *FGFR* rearrangement were most frequently represented (cholangiocarcinoma: 14/22 [63.6%]; NSCLC: 1/12 [8.3%]; esophageal cancer: 1/1 [100%]), followed by those with *FGFR* short variant (cholangiocarcinoma: 8/22 [36.4%]; NSCLC: 4/12 [33.3%]). Seven (58.3%) patients with NSCLC had *RET* rearrangement.

**Table 1:** Summary of patient demographics and baseline characteristics

	Analysis set: treated cholangiocarcinoma patients	Analysis set: treated NSCLC patients			All treated^a^
	8 mg (QD)	8 mg (QD)	10 mg (7 days on/7 days off)	Total	
N	22	8	4	12	35
Age, median (range), years	51.5 (29; 69)	53 (25; 69)	64 (54; 78)	54 (25; 78)	54 (25; 78)
Sex					
Male	13 (59.1%)	5 (62.5%)	3 (75.0%)	8 (66.7%)	22 (62.9%)
Female	9 (40.9%)	3 (37.5%)	1 (25.0%)	4 (33.3%)	13 (37.1%)
Genetic aberrations					
*FGFR* rearrangement	14 (63.6%)	1 (12.5%)	0	1 (8.3%)	16 (45.7%)
*FGFR* short variant	8 (36.4%)	2 (25.0%)	2 (50.0%)	4 (33.3%)	12 (34.3%)
*RET* rearrangement	0	5 (62.5%)	2 (50.0%)	7 (58.3%)	7 (20.0%)
Histology					
Adenocarcinoma	20 (90.9%)	6 (75.0%)	2 (50.0%)	8 (66.7%)	28 (80.0%)
Squamous cell carcinoma	0	1 (12.5%)	1 (25.0%)	2 (16.7%)	3 (8.6%)
Other	2 (9.1%)	1 (12.5%)	1 (25.0%)	2 (16.7%)	4 (11.4%)
Cancer stage at study entry					
IV	22 (100.0%)	8 (100.0%)	4 (100.0%)	12 (100.0%)	35 (100.0%)
Locations of metastatic disease at study entry					
Bone	1 (4.5%)	1 (12.5%)	0	1 (8.3%)	2 (5.7%)
Brain	0	1 (12.5%)	0	1 (8.3%)	1 (2.9%)
Liver	8 (36.4%)	0	1 (25.0%)	1 (8.3%)	9 (25.7%)
Lung	8 (36.4%)	2 (25.0%)	0	2 (16.7%)	11 (31.4%)
Other	5 (22.7%)	4 (50.0%)	3 (75.0%)	7 (58.3%)	12 (34.3%)
Prior systemic therapy					
Biological agents	1 (4.5%)	1 (12.5%)	0	1 (8.3%)	2 (5.7%)
Chemotherapy	22 (100.0%)	8 (100.0%)	4 (100.0%)	12 (100.0%)	35 (100%)
Immunotherapy	0	2 (25.0%)	1 (25.0%)	3 (25.0%)	3 (8.6%)
ECOG PS					
0	12 (54.5%)	4 (50.0%)	0	4 (33.3%)	16 (45.7%)
1	10 (45.5%)	4 (50.0%)	4 (100.0%)	8 (66.7%)	19 (54.3%)

Note: Data are *n* (%) unless otherwise stated. Percentages are calculated with the number of patients with non-missing values of each parameter of each group as the denominator

ECOG PS, Eastern Cooperative Oncology Group performance status; *FGFR*, fibroblast growth factor receptor; NSCLC, non-small cell lung cancer; QD, once daily; *RET*, RET proto-oncogene

^a^Includes the only metastatic esophageal cancer patient, a 56-year-old male with *FGFR3* rearrangement. He received prior radiotherapy, prior cancer-related surgery, and systemic therapy before study treatment

### Efficacy

All 35 patients were included in the efficacy analysis. The median treatment duration for all patients was 3.8 (range, 0.5–35.6) months (cholangiocarcinoma: 6.2 [range, 1.5–35.6] months; NSCLC: 1.7 [range, 0.5–9.4] months; esophageal cancer: treatment duration was 10.4 months).

**Fig. 1:**
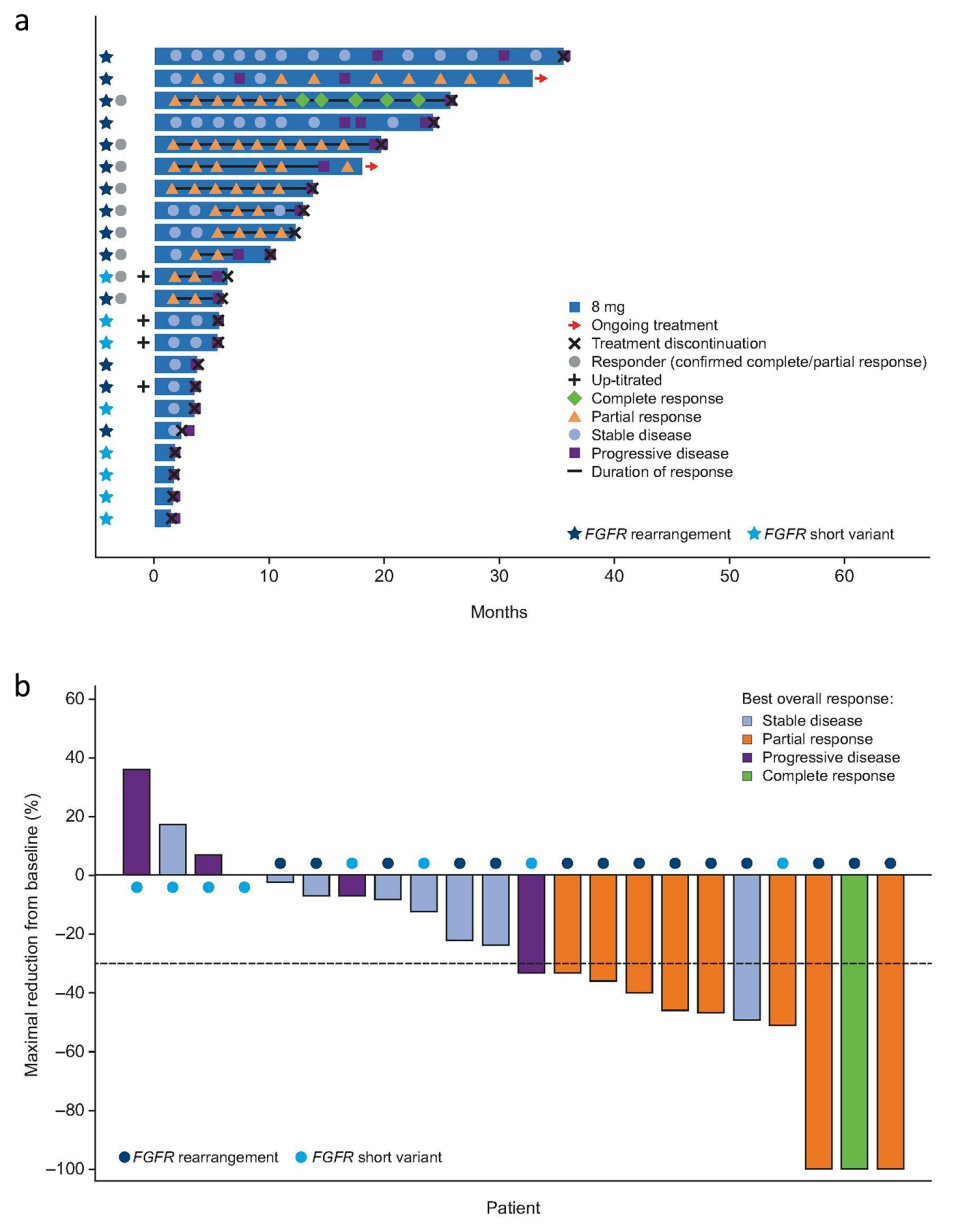
**a**) Swim lane plot for treatment duration and response and **b**) maximal percentage reduction of sum of target lesion diameters from baseline for CCA (*N* = 22)

**Fig. 2:**
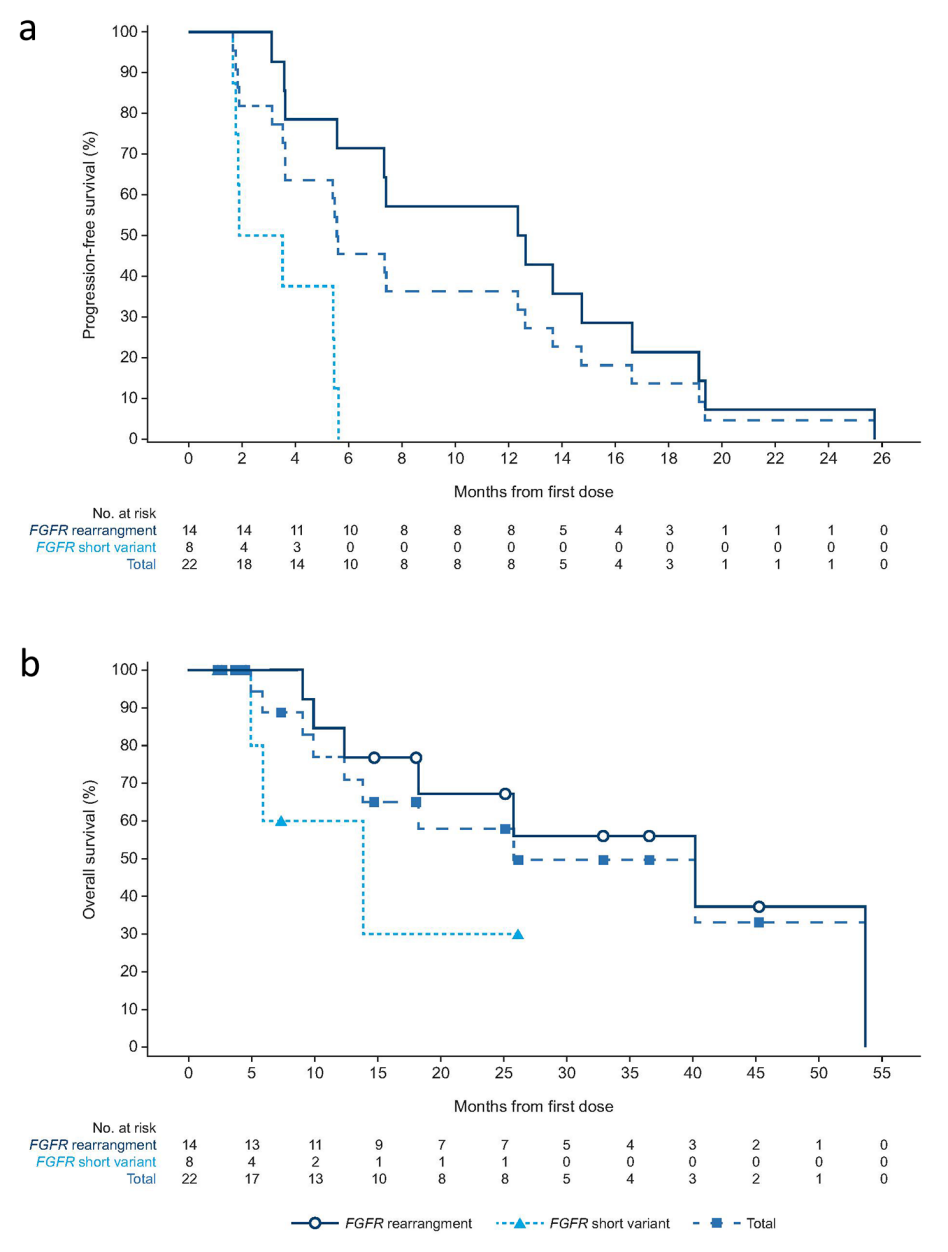
Kaplan–Meier plots for **a**) progression-free survival and **b**) overall survival in patients with cholangiocarcinoma. The symbols on the curve represent censoring

The confirmed investigator-assessed ORR was 40.9% (95% CI, 20.7–63.6) for all patients with cholangiocarcinoma (Table 2); one (4.5%) patient achieved complete response (CR) and eight (36.4%) achieved partial response (PR). ORR for those with *FGFR* rearrangement was 57.1% (95% CI, 28.9–82.3) and 12.5% (95% CI, 0.3–52.7) for those with *FGFR* short variant. Median DOR was 7.3 (range, 3.7–17.5) months (Fig. 1a). The waterfall plot showing the sum of target lesion size is presented in Fig. 1b. The median PFS was 5.6 months (95% CI, 3.6–12.7) and median OS was 25.8 months (95% CI, 9.9–not estimable [NE]) (Fig. 2). Based on the efficacy results, the primary endpoint of the cholangiocarcinoma group was met as the ORR lower boundary of the CI was more than 15%.

**Table 2:** Investigator-assessed efficacy data by tumor type

	Cholangiocarcinoma^a^			NSCLC		
	*FGFR* rearrangement	*FGFR* short variant	Total	8 mg (QD)	10 mg (7 days on/7 days off)	Total
Analysis set: treated patients	14	8	22	8	4	12
Objective response rate^b^ 95% CI^c^	8 (57.1) (28.9–82.3)	1 (12.5) (0.3–52.7)	9 (40.9) (20.7–63.6)	0 NE	0 NE	0 NE
Disease control rate^d^ 95% CI^c^	14 (100.0) (76.8–100.0)	4 (50.0) (15.7–84.3)	18 (81.8) (59.7–94.8)	2 (25.0) (3.2–65.1)	1 (25.0) (0.6–80.6)	3 (25.0) (5.5–57.2)
Best overall response						
Complete response Partial response Stable disease Progressive disease Not evaluable	1 (7.1) 7 (50.0) 6 (42.9) 0 0	0 1 (12.5) 3 (37.5) 4 (50.0) 0	1 (4.5) 8 (36.4) 9 (40.9) 4 (18.2) 0	0 0 2 (25.0) 6 (75.0) 0	0 0 1 (25.0) 2 (50.0) 1 (25.0)	0 0 3 (25.0) 8 (66.7) 1 (8.3)
PFS^e^						
Median, months 95% CI	12.5 (3.7–16.6)	2.7 (1.7–5.5)	5.6 (3.6–12.7)	1.6 (0.72–NE)	1.9 (1.8–NE)	1.8 (0.8–1.9)
6-month PFS rate, % 95% CI	71 (41–88)	0 (NE–NE)	45 (24–64)	0 (NE–NE)	33 (< 1–77)	11 (< 1–37)
12-month PFS rate, % 95% CI	57 (28–78)	0 (NE–NE)	36 (17–56)	0 (NE–NE)	0 (NE–NE)	0 (NE–NE)
OS^e^						
Median, months 95% CI 6-month OS rate, % 95% CI 12-month OS rate, % 95% CI	40.2 (12.4–NE) 100 (100–100) 85 (51–96)	13.8 (4.9–NE) 60 (13–88) 60 (13–88)	25.8 (9.9–NE) 89 (62–97) 77 (50–91)	NE (NE–NE) 100 (100–100) NE (NE–NE)	NE (NE–NE) 100 (100–100) NE (NE–NE)	NE (NE–NE) 100 (100–100) NE (NE–NE)

Note: Data are *n* (%) unless otherwise stated. Complete response and partial response must be confirmed by repeat assessments ≥ 4 weeks from the initial observation. For a response to qualify as stable disease, follow-up measurements must have met the stable disease criteria at least once at a minimum interval not less than 6 weeks after the first dose of study agent

CI, confidence interval; *FGFR*, fibroblast growth factor receptor; NE, not estimable; NSCLC, non-small cell lung cancer; PFS, progression-free survival; OS, overall survival; QD, once daily

^a^All patients with cholangiocarcinoma received erdafitinib 8 mg QD. ^b^Objective response rate is defined as patients with complete response or partial response to treatment. ^c^95% CI is based on the Clopper–Pearson method. ^d^Disease control rate is defined as patients with complete response, partial response, and stable disease after treatment. ^e^Based on Kaplan–Meier estimate

The treated esophageal cancer patient not listed in this table achieved partial response. Investigator-assessed PFS was 10.2 months. Survival time was more than 10.4 months

**Fig. 3:**
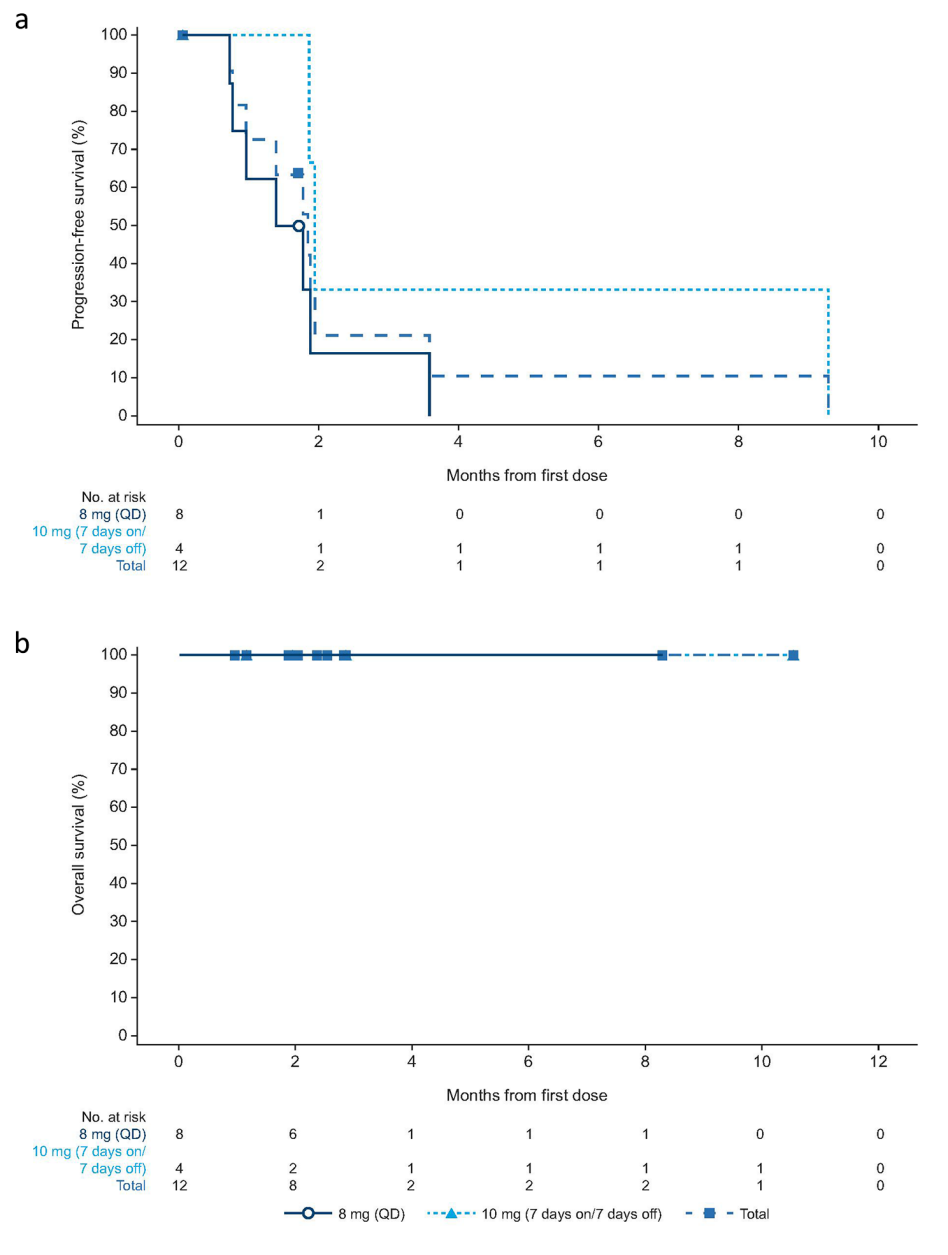
**a**) Kaplan–Meier plots for progression-free survival and **b**) overall survival in patients with NSCLC. The symbols on the curve represent censoring

No patients with NSCLC achieved CR or PR (Table 2). The DCR was 25.0% (95% CI, 5.5–57.2%). The median PFS was 1.8 months (95% CI, 0.8–1.9; Fig. 3a). No death was captured, and median OS was NE (Fig. 3b). Three patients with NSCLC achieved stable disease and two experienced tumor shrinkage of more than − 20% (Supplementary Figure S2).

PR was achieved for the single case of esophageal cancer; PFS was 10.2 months and DOR was 8.5 months.

### Safety

All patients experienced at least one treatment-emergent AE (TEAE), and grade ≥ 3 TEAEs were reported in 22 (62.9%) patients (Table 3). Serious AEs occurred in 16 (45.7%) patients, which were drug related for three (8.6%). The three drug-related serious AEs (pneumonia, alanine aminotransferase increased, rash erythematous) led to dose interruption and were eventually resolved. Hyperphosphatemia was the most frequently reported TEAE (*n* = 30, 85.7%). There were 35 cases of prolonged hyperphosphatemia, eight had phosphate levels of ≥ 5.5 mg/dL for > 1 month, and only one had anemia which was considered a potential sequalae of prolonged hyperphosphatemia. Other TEAEs that occurred with a frequency of ≥ 30% were dry mouth (*n* = 18, 51.4%), stomatitis (*n* = 17, 48.6%), alanine aminotransferase increased (*n* = 16, 45.7%), aspartate aminotransferase increased (*n* = 15, 42.9%), diarrhea (*n* = 13, 37.1%), decreased appetite (*n* = 12, 34.3%), dry skin (*n* = 12, 34.3%), and constipation (*n* = 11, 31.4%). TEAEs related to erdafitinib were managed by dose interruptions, dose reductions, and supportive care.

**Table 3:** TEAEs for the total population

Summary of TEAEs	8 mg (QD)	10 mg (7 days on/7 days off)	Total
Analysis set: treated patients	31	4	35
Any AE	31 (100.0)	4 (100.0)	35 (100.0)
Grade 3 or worse	20 (64.5)	2 (50.0)	22 (62.9)
Any drug-related^a^ AE	31 (100.0)	2 (50.0)	33 (94.3)
Grade 3 or worse	13 (41.9)	0	13 (37.1)
Any serious AE	15 (48.4)	1 (25.0)	16 (45.7)
Drug-related^a^	3 (9.7)	0	3 (8.6)
AE leading to treatment discontinuation	2 (6.5)	0	2 (5.7)
AE leading to treatment interruption	28 (90.3)	2 (50.0)	30 (85.7)
AE leading to treatment reduction	19 (61.3)	0	19 (54.3)
AE leading to death	1 (3.2)	0	1 (2.9)
TEAEs occurring in ≥ 20% of patients (all grades) by preferred term			
Hyperphosphatemia	29 (93.5)	1 (25.0)	30 (85.7)
Decreased appetite	10 (32.3)	2 (50.0)	12 (34.3)
Dry mouth	18 (58.1)	0	18 (51.4)
Stomatitis	16 (51.6)	1 (25.0)	17 (48.6)
Diarrhea	12 (38.7)	1 (25.0)	13 (37.1)
Constipation	10 (32.3)	1 (25.0)	11 (31.4)
Dry skin	12 (38.7)	0	12 (34.3)
Nail discoloration	9 (29.0)	1 (25.0)	10 (28.6)
Nail loss	7 (22.6)	2 (50.0)	9 (25.7)
Palmar-plantar erythrodysesthesia syndrome	7 (22.6)	0	7 (20.0)
Paronychia	6 (19.4)	1 (25.0)	7 (20.0)
Alanine aminotransferase increased	15 (48.4)	1 (25.0)	16 (45.7)
Aspartate aminotransferase increased	14 (45.2)	1 (25.0)	15 (42.9)
Dry eye	8 (25.8)	0	8 (22.9)
Dysgeusia	7 (22.6)	0	7 (20.0)

Note: Data are *n* (%). Percentages calculated with the number of patients in the all-treated population of each group as the denominator. Recurring events are counted only once for each patient

AE, adverse event; QD, once daily; TEAE, treatment-emergent adverse event

^a^Adverse events reported as possible, probable, or very likely related to the study drug are classified as related

Drug-related TEAEs were reported for 33 (94.3%) patients. The most frequently occurring drug-related TEAEs were hyperphosphatemia (*n* = 30, 85.7%), followed by dry mouth (*n* = 18, 51.4%) and stomatitis (*n* = 17, 48.6%) (Supplementary Table S3). Grade ≥ 3 drug-related TEAEs were reported in 13 (37.1%) patients. Detachment of retinal pigment epithelium was reported in one patient on erdafitinib 8 mg QD and was the only TEAE of special interest.

TEAEs leading to dose reduction occurred in 19 (54.3%) patients, with nail disorder (*n* = 4, 11.4%), hyperphosphatemia (*n* = 4, 11.4%), and stomatitis (*n* = 4, 11.4%) most commonly reported; 18 were considered related to erdafitinib. TEAEs leading to treatment discontinuation occurred in two (5.7%, arthralgia and rash erythematous) patients and were considered related to treatment. One (2.9%) patient died due to an AE (sepsis), which was considered not related to erdafitinib.

### Pharmacokinetics

Mean plasma concentrations of erdafitinib were measured for both 8 mg QD and 10 mg 7 days on/7 days off regimens (Supplementary Table S4). For those on the 8 mg QD dosing schedule, at 3 and 6 h post-dose, corresponding approximately to the maximum serum concentration, the concentrations were 354 and 340 ng/mL, respectively, on cycle 1 day 1, and were 1,026 and 1,007 ng/mL, respectively, on cycle 1 day 14. In the 10 mg 7 days on/7 days off regimen, the mean plasma concentrations of erdafitinib at 3 and 6 h post-dose were 355 and 407 ng/mL, respectively, on cycle 1 day 1, and were 1,371 and 1,433 ng/mL, respectively, on cycle 1 day 7 for those remaining on the treatment regimen. Mean fraction of erdafitinib unbound to plasma protein was 0.325 ± 0.157%; the corresponding mean AGP concentration was 78.0 ± 47.4 mg/dL.

## Discussion

This study assessed the efficacy and safety of the selective pan-FGFR inhibitor erdafitinib in Asian patients with advanced cholangiocarcinoma, NSCLC, and esophageal cancer. Objective responses were seen in nine (40.9%) patients with cholangiocarcinoma, with one and eight CR and PRs, respectively. Tumor shrinkage was observed in more than 50% of all patients with cholangiocarcinoma. Notably, ORR was higher among those with *FGFR* rearrangement than short variant. However, no objective responses were documented in patients with NSCLC, including those with *RET* alterations, with three (25%) patients experiencing disease stabilization as best response. Promising antitumor activity was also observed in esophageal cancer as the only patient with this disease enrolled in this study achieved PR as best response. Most of the TEAEs and drug-related TEAEs were generally of grade 1 or 2 in severity and were manageable by adequate dose modification. There were no treatment-related deaths due to TEAEs recorded.

Cross-trial comparisons should be interpreted with caution given the differences in study design, dosing schedules, and patient populations enrolled. In phase I and II studies of other FGFR inhibitors [10, 12, 13, 29], pemigatinib ORR was 35.5% (95% CI, 26.5–45.4), infigratinib ORR was 23.1% (95% CI, 15.6–32.2), and futibatinib was 41.7% (95% CI, 32.1–51.9) [10, 12, 29]. However, the lower ORR observed in some of these studies could be attributed to the larger proportion of heavily pretreated patients enrolled [10, 12]. In terms of survival outcomes, among patients receiving erdafitinib, the median PFS was 5.6 months (95% CI, 3.6–12.7) and the median OS was 25.8 months (95% CI, 9.9–NE). However, the OS results should be interpreted with caution as six patients were lost to follow-up following disease progression and were censored in the OS curve (Fig. 2b). In other advanced/metastatic *FGFR2* fusion or rearrangement-positive cholangiocarcinoma studies, pemigatinib reported a median PFS of 6.9 months (95% CI, 6.2–9.6) and OS of 21.1 months (95% CI, 14.8–NE) [10], infigratinib reported a median PFS of 7.3 months (95% CI, 5.6 − 7.6) and OS of 12.2 months (95% CI, 10.7 − 14.9) [12], and futibatinib reported a median PFS of 5.1 months (95% CI, 3.7–9.0) [13]. Of note, ORR in the current erdafitinib study was relatively higher than an earlier phase I study for advanced cholangiocarcinoma (40.9% vs. 27.0%), in which the majority (*n* = 10/11) of patients were treated with an intermittent schedule of erdafitinib 10 mg for 7 days on/7 days off [30].

*RET*-rearranged NSCLC patients were included in this study based on pre-clinical evidence that erdafitinib, a small molecular kinase inhibitor, displayed high selectivity with a binding affinity of 1.88 nM to *RET*, lower than the binding affinity with the four different members of *FGFR* (unpublished data). Given that *RET* rearrangement is a driver alteration in lung adenocarcinoma [31], occurring in approximately 1–2% of NSCLC [32], they were included as an eligible molecular biomarker in our study. However, no patients in the NSCLC cohort achieved CR or PR. This result could be partially explained by the diverse biomarker profile of patients recruited in the study (seven *RET* fusions, four *FGFR* mutations, and one *FGFR3* fusion) and the biological complexity of lung adenocarcinoma, which makes up the majority of the NSCLC cohort. Conversely, in the RAGNAR study, interim results showed that responses were reported in both *FGFR1–4*-altered squamous (*n* = 3/11, 27.3%) and non-squamous (*n* = 1/7, 14.3%) NSCLC who were treated with erdafitinib [27], suggesting that FGFR inhibitors may have a role to play in NSCLC.

Our findings are intriguing in the single male patient with esophageal cancer who had received prior systemic therapy. There is little previously reported evidence on the clinical benefit of FGFR inhibitors in esophageal cancer. Further studies are warranted given that *FGFR1* overexpression has been implicated in the poor prognosis of esophageal squamous cell carcinoma [19].

Although limited by sample size, our pharmacokinetic analysis of erdafitinib in Asian patients suggested that plasma concentrations were similar to those from an earlier phase I trial of erdafitinib in patients with advanced solid tumors at corresponding time points [30]. This trend was also generally similar to that of an earlier study in Japanese patients where the time taken for erdafitinib to reach maximum concentration was around 2–3 h after the first dose on day 1 and 2–6 h in subsequent daily doses [33]. The time to maximum concentration in Caucasian adult patients from the United States and Europe was between 2 and 4 h with a long half-life ranging from 50 to 60 h [34].

The safety profile of erdafitinib was acceptable and consistent with previous observations in non-Asian populations, with no unexpected safety signals identified in either dosing regimens [25–27, 30]. In this study, all patients experienced TEAEs, of which 62.9% were grade ≥ 3 in severity. Hyperphosphatemia and gastrointestinal toxicity were the most common TEAEs, the former is a known on-target toxicity of FGFR inhibitors [4, 30, 35]. Drug-related TEAEs were reported in 94.3% of patients, 37.1% of which were grade ≥ 3 in severity. These rates were generally consistent with an earlier phase I trial of erdafitinib in patients with advanced solid tumors [30]. The safety profile of erdafitinib was also comparable to that of other selective FGFR inhibitors in solid tumors [12, 13, 35, 36].

This is the first study on erdafitinib to enroll patients from East Asia, providing valuable insights on Asian patients with *FGFR*-altered advanced solid tumors outside of urothelial carcinoma. The limitations of this study include the limited sample size in each tumor cohort and the open-label, single-arm study design. The responses and safety profile observed in each of the present tumor cohorts need to be verified in larger populations and in randomized controlled studies. The small sample size also made it difficult to assess treatment efficacy among different *FGFR* alterations. However, in the RAGNAR study, comparable efficacy was achieved with erdafitinib regardless of the type of *FGFR* alterations in advanced solid tumors [27]. It was also not possible to determine the degree of antitumor activity based on the location of the tumor in cholangiocarcinoma, given that the marked difference in characteristics between intrahepatic and extrahepatic tumors could be associated with different treatment outcomes.

## Conclusion

In conclusion, erdafitinib was well tolerated with a manageable safety profile in patients with advanced solid tumors. Erdafitinib also showed promising efficacy in the select advanced solid tumors, and particularly had a durable effect in Asian patients with advanced *FGFR*-altered cholangiocarcinoma. These data support the continued evaluation of erdafitinib in patients with advanced solid tumors and *FGFR* aberrations in the ongoing multinational phase II RAGNAR study.

### Electronic supplementary material

Below is the link to the electronic supplementary material.

Supplementary Material 1

## Data Availability

The data sharing policy of Johnson & Johnson Innovative Medicine is available at https://www.janssen.com/clinical-trials/transparency. As noted on this site, requests for access to the study data can be submitted through Yale Open Data Access (YODA) Project site at http://yoda.yale.edu.

## Abbreviations

AGP: Alpha-1-acid glycoprotein
AE: Adverse event
CI: Confidence interval
CR: Complete response
CT: Computed tomography
DCR: Disease control rate
DOR: Duration of response
ECOG PS: Eastern Cooperative Oncology Group performance status
FGFR: Fibroblast growth factor receptor
MedDRA: Medical Dictionary for Regulatory Activities
NCI-CTCAE v4.0: National Cancer Institute Common Terminology Criteria for Adverse Events Version 4.0
NE: Not estimable
NSCLC: Non-small cell lung cancer
ORR: Objective response rate
OS: Overall survival
PFS: Progression-free survival
PR: Partial response
QD: Twice a day
RET: RET proto-oncogene
RECIST v1.1: Response Evaluation Criteria in Solid Tumors version 1.1
TEAE: Treatment-emergent adverse event

## References

[1] Krook MA, Reeser JW, Ernst G, Barker H, Wilberding M, Li G, et al. Fibroblast growth factor receptors in cancer: genetic alterations, diagnostics, therapeutic targets and mechanisms of resistance. Br J Cancer. 2021;124:880–92. 33268819 10.1038/s41416-020-01157-0 PMC7921129

[2] Sleeman M, Fraser J, McDonald M, Yuan S, White D, Grandison P, et al. Identification of a new fibroblast growth factor receptor, FGFR5. Gene. 2001;271:171–82. 11418238 10.1016/S0378-1119(01)00518-2

[3] Babina IS, Turner NC. Advances and challenges in targeting FGFR signalling in cancer. Nat Rev Cancer. 2017;17:318–32. 28303906 10.1038/nrc.2017.8

[4] Goyal L, Kongpetch S, Crolley VE, Bridgewater J. Targeting FGFR inhibition in cholangiocarcinoma. Cancer Treat Rev. 2021;95:102170. 33735689 10.1016/j.ctrv.2021.102170

[5] Zuo W, He Y, Li W, Wu H, Zhao Z, Zhang Y, et al. Landscape of FGF/FGFR alterations in 12,372 Chinese Cancer patients. J Cancer. 2020;11:6695–9. 33046990 10.7150/jca.49269 PMC7545692

[6] Zhou Z, Liu Z, Ou Q, Wu X, Wang X, Shao Y, et al. Targeting FGFR in non-small cell lung cancer: implications from the landscape of clinically actionable aberrations of FGFR kinases. Cancer Biol Med. 2021;8:490–501. 10.20892/j.issn.2095-3941.2020.0120 PMC8185861 33710807

[7] Sung H, Ferlay J, Siegel RL, Laversanne M, Soerjomataram I, Jemal A, et al. Global Cancer statistics 2020: GLOBOCAN estimates of incidence and Mortality Worldwide for 36 cancers in 185 countries. CA Cancer J Clin. 2021;71:209–49. 33538338 10.3322/caac.21660

[8] Banales JM, Marin JJG, Lamarca A, Rodrigues PM, Khan SA, Roberts LR, et al. Cholangiocarcinoma 2020: the next horizon in mechanisms and management. Nat Rev Gastroenterol Hepatol. 2020;17:557–88. 32606456 10.1038/s41575-020-0310-z PMC7447603

[9] Lamarca A, Palmer DH, Wasan HS, Ross PJ, Ma YT, Arora A, et al. Second-line FOLFOX chemotherapy versus active symptom control for advanced biliary tract cancer (ABC-06): a phase 3, open-label, randomised, controlled trial. Lancet Oncol. 2021;22:690–701. 33798493 10.1016/S1470-2045(21)00027-9 PMC8082275

[10] Abou-Alfa GK, Sahai V, Hollebecque A, Vaccaro G, Melisi D, Al-Rajabi R, et al. Pemigatinib for previously treated, locally advanced or metastatic cholangiocarcinoma: a multicentre, open-label, phase 2 study. Lancet Oncol. 2020;21:671–84. 32203698 10.1016/S1470-2045(20)30109-1 PMC8461541

[11] Eisenhauer EA, Therasse P, Bogaerts J, Schwartz LH, Sargent D, Ford R, et al. New response evaluation criteria in solid tumours: revised RECIST guideline (version 1.1). Eur J Cancer. 2009;45:228–47. 19097774 10.1016/j.ejca.2008.10.026

[12] Javle M, Roychowdhury S, Kelley RK, Sadeghi S, Macarulla T, Weiss KH, et al. Infigratinib (BGJ398) in previously treated patients with advanced or metastatic cholangiocarcinoma with FGFR2 fusions or rearrangements: mature results from a multicentre, open-label, single-arm, phase 2 study. Lancet Gastroenterol Hepatol. 2021;6:803–15. 34358484 10.1016/S2468-1253(21)00196-5

[13] Meric-Bernstam F, Bahleda R, Hierro C, Sanson M, Bridgewater J, Arkenau HT, et al. Futibatinib, an irreversible FGFR1-4 inhibitor, in patients with Advanced Solid tumors harboring FGF/FGFR aberrations: a phase I dose-expansion study. Cancer Discov. 2022;12:402–15. 34551969 10.1158/2159-8290.CD-21-0697 PMC9762334

[14] Ettinger DS, Wood DE, Aisner DL, Akerley W, Bauman JR, Bharat A, et al. NCCN guidelines Insights: Non-small Cell Lung Cancer, Version 2.2021. J Natl Compr Canc Netw. 2021;19:254–66. 33668021 10.6004/jnccn.2021.0013

[15] Heo JY, Yoo SH, Suh KJ, Kim SH, Kim YJ, Ock CY, et al. Clinical pattern of failure after a durable response to immune check inhibitors in non-small cell lung cancer patients. Sci Rep. 2021;11:2514. 33510255 10.1038/s41598-021-81666-x PMC7844257

[16] Nogova L, Sequist LV, Perez Garcia JM, Andre F, Delord JP, Hidalgo M, et al. Evaluation of BGJ398, a fibroblast growth factor receptor 1–3 kinase inhibitor, in patients with Advanced Solid tumors Harboring genetic alterations in fibroblast growth factor receptors: results of a global phase I, dose-escalation and dose-expansion study. J Clin Oncol. 2017;35:157–65. 27870574 10.1200/JCO.2016.67.2048 PMC6865065

[17] Schuler M, Cho BC, Sayehli CM, Navarro A, Soo RA, Richly H, et al. Rogaratinib in patients with advanced cancers selected by FGFR mRNA expression: a phase 1 dose-escalation and dose-expansion study. Lancet Oncol. 2019;20:1454–66. 31405822 10.1016/S1470-2045(19)30412-7

[18] Schuler MH, Tabernero J, Carranza O, Loriot Y, Pant S, Arnold D, et al. Efficacy and safety of erdafitinib in adults with NSCLC and prespecified fibroblast growth factor receptor alterations in the phase 2 open-label, single-arm RAGNAR trial. J Clin Oncol. 2024;42:8515. 10.1200/JCO.2024.42.16_suppl.8515

[19] Chen B, Liu S, Gan L, Wang J, Hu B, Xu H, et al. FGFR1 signaling potentiates tumor growth and predicts poor prognosis in esophageal squamous cell carcinoma patients. Cancer Biol Ther. 2018;19:76–86. 29257923 10.1080/15384047.2017.1394541 PMC5790345

[20] Wainberg ZA, Enzinger PC, Kang YK, Qin S, Yamaguchi K, Kim IH, et al. Bemarituzumab in patients with FGFR2b-selected gastric or gastro-oesophageal junction adenocarcinoma (FIGHT): a randomised, double-blind, placebo-controlled, phase 2 study. Lancet Oncol. 2022;23:1430–40. 36244398 10.1016/S1470-2045(22)00603-9

[21] U.S. FDA. FDA grants accelerated approval to futibatinib for cholangiocarcinoma. https://www.fda.gov/drugs/resources-information-approved-drugs/fda-grants-accelerated-approval-futibatinib-cholangiocarcinoma. Accessed Jan 2023.

[22] U.S. FDA. FDA grants accelerated approval to infigratinib for metastatic cholangiocarcinoma. https://www.fda.gov/drugs/resources-information-approved-drugs/fda-grants-accelerated-approval-infigratinib-metastatic-cholangiocarcinoma. Accessed Jan 2023.

[23] U.S. FDA. FDA grants accelerated approval to pemigatinib for cholangiocarcinoma with an FGFR2 rearrangement or fusion. https://www.fda.gov/drugs/resources-information-approved-drugs/fda-grants-accelerated-approval-pemigatinib-cholangiocarcinoma-fgfr2-rearrangement-or-fusion. Accessed Jan 2023.

[24] BALVERSA Prescribing Information. https://www.accessdata.fda.gov/drugsatfda_docs/label/2019/212018s000lbl.pdf. Accessed Aug 2023.

[25] Loriot Y, Necchi A, Park SH, Garcia-Donas J, Huddart R, Burgess E, et al. Erdafitinib in locally Advanced or Metastatic Urothelial Carcinoma. N Engl J Med. 2019;381:338–48. 31340094 10.1056/NEJMoa1817323

[26] Siefker-Radtke AO, Necchi A, Park SH, Garcia-Donas J, Huddart RA, Burgess EF, et al. Efficacy and safety of erdafitinib in patients with locally advanced or metastatic urothelial carcinoma: long-term follow-up of a phase 2 study. Lancet Oncol. 2022;23:248–58. 35030333 10.1016/S1470-2045(21)00660-4

[27] Loriot Y, Schuler MH, Iyer G, Witt O, Doi T, Qin S, et al. Tumor agnostic efficacy and safety of erdafitinib in patients (pts) with advanced solid tumors with prespecified fibroblast growth factor receptor alterations (FGFRalt) in RAGNAR: interim analysis (IA) results. J Clin Oncol. 2022;40:3007. 10.1200/JCO.2022.40.16_suppl.3007

[28] Loriot Y, Matsubara N, Park SH, Huddart RA, Burgess EF, Houede N, et al. Erdafitinib or Chemotherapy in Advanced or Metastatic Urothelial Carcinoma. N Engl J Med. 2023;389:1961–71. 37870920 10.1056/NEJMoa2308849

[29] Goyal L, Meric-Bernstam F, Hollebecque A, Morizane C, Valle JW, Karasic TB, et al. Updated results of the FOENIX-CCA2 trial: efficacy and safety of futibatinib in intrahepatic cholangiocarcinoma (iCCA) harboring FGFR2 fusions/rearrangements. J Clin Oncol. 2022;40:4009. 10.1200/JCO.2022.40.16_suppl.4009

[30] Bahleda R, Italiano A, Hierro C, Mita A, Cervantes A, Chan N, et al. Multicenter Phase I study of Erdafitinib (JNJ-42756493), oral pan-fibroblast growth factor receptor inhibitor, in patients with Advanced or Refractory Solid tumors. Clin Cancer Res. 2019;25:4888–97. 31088831 10.1158/1078-0432.CCR-18-3334

[31] Takeuchi K, Soda M, Togashi Y, Suzuki R, Sakata S, Hatano S, et al. RET, ROS1 and ALK fusions in lung cancer. Nat Med. 2012;18:378–81. 22327623 10.1038/nm.2658

[32] Kato S, Subbiah V, Marchlik E, Elkin SK, Carter JL, Kurzrock R. RET aberrations in diverse cancers: next-generation sequencing of 4,871 patients. Clin Cancer Res. 2017;23:1988–97. 27683183 10.1158/1078-0432.CCR-16-1679

[33] Nishina T, Takahashi S, Iwasawa R, Noguchi H, Aoki M, Doi T. Safety, pharmacokinetic, and pharmacodynamics of erdafitinib, a pan-fibroblast growth factor receptor (FGFR) tyrosine kinase inhibitor, in patients with advanced or refractory solid tumors. Invest New Drugs. 2018;36:424–34. 28965185 10.1007/s10637-017-0514-4

[34] Tabernero J, Bahleda R, Dienstmann R, Infante JR, Mita A, Italiano A, et al. Phase I dose-escalation study of JNJ-42756493, an oral pan-fibroblast growth factor receptor inhibitor, in patients with Advanced Solid tumors. J Clin Oncol. 2015;33:3401–8. 26324363 10.1200/JCO.2014.60.7341

[35] Bahleda R, Meric-Bernstam F, Goyal L, Tran B, He Y, Yamamiya I, et al. Phase I, first-in-human study of futibatinib, a highly selective, irreversible FGFR1-4 inhibitor in patients with advanced solid tumors. Ann Oncol. 2020;31:1405–12. 32622884 10.1016/j.annonc.2020.06.018 PMC9743148

[36] Voss MH, Hierro C, Heist RS, Cleary JM, Meric-Bernstam F, Tabernero J, et al. A phase I, Open-Label, Multicenter, dose-escalation study of the oral selective FGFR inhibitor Debio 1347 in patients with advanced solid tumors harboring FGFR gene alterations. Clin Cancer Res. 2019;25:2699–707. 30745300 10.1158/1078-0432.CCR-18-1959 PMC9014845

