# Supplementary material for: Erdafitinib in Asian patients with advanced solid tumors: an open-label, single-arm, phase IIa trial
Source: BMC Cancer. 2024 Aug 13;24:1006. doi: 10.1186/s12885-024-12584-0 (PMC11323360; doi:10.1186/s12885-024-12584-0)
Supplement: Supplementary file 1 — Supplementary Material 1 [file 12885_2024_12584_MOESM1_ESM.docx]

# Supplementary Appendix

**Supplementary Table S1. Study eligibility criteria**

| **Inclusion criteria** |
| --- |
| 1. Subjects must be 18 (or the legal age of consent in the jurisdiction in which the study is taking place) years of age or older; |
| 1. Pathologically or cytologically confirmed, advanced or refractory tumors (there are no restriction on the total number of lines of prior therapies, but patient should have received after at least 1 line of anti-cancer therapy [as per local standard of care]):    - Cohort A: Squamous and non-squamous NSCLC    - Cohort B: Esophageal cancer, urothelial cancer, and cholangiocarcinoma; |
| 1. Subjects must meet the following molecular eligibility criteria (diagnosed at a central or local laboratory using tumor tissue-based assay, which must indicate at least 1 of the following):    - *FGFR* gene translocations such as, but not limited to: *FGFR2-BICC1*, *FGFR2- CASP7*, *FGFR3-TACC3*, *FGFR3-BAIAP2L1*, *FGFR2-AFF3*, *FGFR2-OFD1*, *FGFR1-TACC1*, *FGFR2-TACC2*, *FGFR2-PPAPDC1A*, *FGFR2-SLC45A3*, *FGFR2-VCL*, *FGFR2-CCDC147*, *FGFR2-CCAR1*, *FGFR2-UBQLN1*, *FGFR2-MCU*, *FGFR3-WHSC1*, *FGFR2-CCDC6*    - *FGFR* gene mutations such as, but not limited to: *FGFR1*: *T141A*, *N546K*, *K656E*; *FGFR2*: *S252W*, *P253R*, *Y375C*, *G380R*, *C382R*, *N549K*, *K659E*; *FGFR3*: *R248C*, *S249C*, *G370C*, *S371C*, *Y373C*, *F384L*, *A391E*, *K650E*, *K650M*, *K650Q*, *K650T*, *G697C*    - *RET* activating mutations or *RET* translocations    - Subjects with evidence of FGFR pathway activation or other potential target/pathway inhibited by erdafitinib may also be considered and allowed for enrollment if supported by emerging biomarker data; |
| 1. The presence of measurable disease according to the RECIST, Version 1.1 Criteria, and documented disease progression as defined by RECIST (Version 1.1) at baseline; |
| 1. Eastern Cooperative Oncology Group (ECOG) performance status score 0 or 1; |
| 1. Adequate bone marrow, liver, and renal function within the 14 days prior to Day 1 of Cycle 1 up until pre-dose of Cycle 1, as described below:    - Bone marrow function (without the support of cytokines and/or erythropoietin in preceding 2 weeks):      - Absolute neutrophil count (ANC) >1500/mm^3^      - Platelet count >75,000/mm^3^      - Hemoglobin >8.5 g/dL (without transfusion or demonstrate stability (ie, No significant decline in hemoglobin for 2 weeks after transfusion)    - Liver function:      - Total bilirubin ≤1.5 x institutional ULN, unless known to have Gilbert's disease      - Alanine aminotransferase and AST ≤2.5 x the institutional ULN (For cholangiocarcinoma, ≤5 x the institutional ULN in case of liver metastasis)    - Renal function:      - Serum creatinine ≤1.5 mg/dl or calculated creatinine clearance (Attachment 2)   ≥50 mL/min/1.73 m^2^; |
| 1. Female subjects (of childbearing potential and sexually active) and male subjects (with a partner of child bearing potential) must use medically acceptable methods of birth control before study entry, for the duration of the study, and for at least 3 months after the last dose of study drug. Male subjects must use highly effective birth control measurements when sexually active and must not donate sperm from the first dose of study drug until 5 months after the last dose of study drug. Medically acceptable methods of contraception that may be used by the subject and/or his/her partner include hormonal prescription oral contraceptives, contraceptive injections, contraceptive patch, intrauterine device, true sexual abstinence, and surgical sterilization (eg, confirmed successful vasectomy or tubal ligation). True sexual abstinence is an acceptable method of contraception and is defined as refraining from heterosexual intercourse during the entire period of the study, including up to 3 months for females and 5 months for males after the last dose of study drug is given. Periodic abstinence (calendar, symptothermal, postovulation methods) is not considered an acceptable contraceptive method; |
| 1. Negative pregnancy test (urine or serum beta human chorionic gonadotropin [β- hCG]) at Screening for women of childbearing potential who are sexually active; |
| 1. Each subject or their legally acceptable representative must sign an informed consent form (ICF) indicating that he or she understands the purpose of and procedures required for the study and are willing to participate in the study. |
| **Exclusion criteria** |
| 1. Chemotherapy, targeted therapies, immunotherapy, or treatment with an investigational anticancer agent within 2 weeks or at least 5 half-lives of the drug, whichever is longer but up to a maximum of 4 weeks, before the first administration of study drug. Localized palliative radiation therapy (but should not include radiation to target lesions) and ongoing luteinizing hormone-releasing hormone (LHRH) agonists, bisphosphonates and denosumab, are permitted; |
| 1. Subjects with persistent phosphate >ULN during screening (within 14 days prior to Day 1 of Cycle 1 up until pre-dose of Cycle 1) and despite medical management of phosphate levels; |
| 1. History or current condition of uncontrolled cardiovascular disease including:    - Unstable angina, myocardial infarction, or congestive heart failure Class II-IV within the preceding 12 months, cerebrovascular accident (CVA), transient ischemic attack (TIA) within the preceding 3 months, pulmonary embolism (PE) within the preceding 2 months    - History of any of the following: sustained ventricular tachycardia, ventricular fibrillation, Torsades de Pointes, cardiac arrest, Mobitz II second degree heart block or third-degree heart block; known presence of dilated, hypertrophic, or restrictive cardiomyopathy    - Obligate use of a cardiac pacemaker or any other cardiac abnormality that, in the opinion of the investigator, medical monitor, or study consultant cardiologist, may place the subject at an unacceptable increased risk with the study drug    - Diagnosed or suspected congenital long QT syndrome    - QTc prolongation as confirmed by triplicate assessment at screening (Fridericia; QTc >450 milliseconds in males or >470 milliseconds in females)    - Family history of short QT syndrome, long QT syndrome; |
| 1. Subjects taking medications known to have a significant risk of causing QTc prolongation and Torsades de Pointes. Subjects who have discontinued any of these medications must have a wash-out period of at least 5 days or at least 5 half-lives of the drug (whichever is longer) prior to the first dose of study drug; |
| 1. Left ventricular ejection fraction (LVEF) <50% as assessed by echocardiography (or multi-gated acquisition [MUGA]) performed at screening; |
| 1. Uncontrolled inter-current illness including, but not limited to, poorly controlled hypertension or diabetes, ongoing active infection requiring antibiotics, psychiatric illness, or at risk of gastrointestinal perforation as per investigators’ assessment; |
| 1. Females who are pregnant, or breast-feeding, or planning to become pregnant and males who plan to father a child while enrolled in this study or within 5 months after the last dose of study drug; |
| 1. Not recovered from reversible toxicity of prior anticancer therapy (except toxicities which are not clinically significant such as alopecia, skin discoloration, or Grade 1 neuropathy).; |
| 1. Any medical condition that requires intact wound healing capacity and is expected to endanger subject safety if wound healing capacity would be severely reduced during administration of the investigational agent (eg, chronic leg ulcers, gastric ulcer disease, skin/decubitus ulcers, or unhealed incisions, or expected major surgery while the investigational agent is being administered); |
| 1. Major surgery within 4 weeks before enrollment; |
| 1. Known human immunodeficiency virus (HIV) infection, or evidence of active hepatitis B or C infection (for example, subjects with history of hepatitis C infection but normal hepatitis C virus polymerase chain reaction test and subjects with hepatitis B with positive hepatitis B surface antigen [HBsAg] antibody are allowed); |
| 1. Active, symptomatic, or untreated brain metastases (subject with prior brain metastases treated at least 3 weeks prior to signing the full-study ICF or that are clinically and radiographically stable for at least 1 month prior to Cycle 1 Day 1 and do not require chronic corticosteroid treatment are allowed to be enrolled); |
| 1. Received prior selective FGFR inhibitor treatment or RET inhibitor treatment, respectively according to the biomarker prescreening result, or the subject has known allergies, hypersensitivity, or intolerance to erdafitinib or its excipients; |
| 1. Exclusion criteria based on ophthalmologic exams:    - History of or current evidence of CSR or retinal vein occlusion (RVO)    - Active wet, age-related macular degeneration (AMD)    - Diabetic retinopathy with macular edema    - Uncontrolled glaucoma (as per local standard of care)    - Corneal pathology such as keratitis, keratoconjunctivitis, keratopathy, corneal    - abrasion, inflammation or ulceration. |

**Supplementary Table S2.** Grading of hyperphosphatemia and skin/nails adverse events.

| **Hyperphosphatemia** |
| --- |
| - Grade 1: 5.5–6.9 mg/dL |
| - Grade 2: 7.0–9.0 mg/dL |
| - Grade 3: 9.0–10 mg/dL, or asymptomatic soft tissue calcification with any phosphate level |
| - Grade 4: >10 mg/dL, or symptomatic soft tissue calcification with any phosphate level |
| **Nail changes (onychodystrophy)** |
| - Grade 1: Nail discoloration, asymptomatic separation of the nail bed from the nail plate or nail loss |
| - Grade 2: Nail/fingertips pain, symptomatic separation of the nail bed from the nail plate or nail loss; limiting instrumental ADL |
| - Grade 3: Severe nail fingertips pain, symptomatic separation of the nail bed from the nail plate or nail loss; significantly limiting instrumental ADL |
| - Grade 4: Not applicable |

**Supplementary Table S3.** Summary of drug-related treatment-related adverse events occurring in ≥20% of patients**.**

| Drug-related TEAEs (all grades) by preferred term, *n* (%) | 8 mg (QD)  *N* = 31 | 10 mg (7 days on/7 days off)  *N* = 4 | Total  *N* = 35 |
| --- | --- | --- | --- |
| Hyperphosphatemia | 29 (93.5%) | 1 (25.0%) | 30 (85.7%) |
| Dry skin | 11 (35.5%) | 0 | 11 (31.4%) |
| Nail discoloration | 9 (29.0%) | 1 (25.0%) | 10 (28.6%) |
| Nail disorder | 7 (22.6%) | 1 (25.0%) | 8 (22.9%) |
| Palmar-plantar erythrodysesthesia syndrome | 7 (22.6%) | 0 | 7 (20.0%) |
| Dry mouth | 18 (58.1%) | 0 | 18 (51.4%) |
| Stomatitis | 15 (48.4%) | 0 | 15 (42.9%) |
| Diarrhea | 8 (25.8%) | 1 (25.0%) | 9 (25.7%) |
| Paronychia | 6 (19.4%) | 1 (25.0%) | 7 (20.0%) |
| Alanine aminotransferase increased | 12 (38.7%) | 0 | 12 (34.3%) |
| Aspartate aminotransferase increased | 11 (35.5%) | 0 | 11 (31.4%) |
| Dry eye | 8 (25.8%) | 0 | 8 (22.9%) |
| Dysgeusia |  |  |  |

Note: Data are *n* (%) unless otherwise stated. Percentages calculated with the number of patients in all-treated population of each group as denominator. Recurring events are counted only once for each patient.

Abbreviations: QD, once daily; TEAE, treatment-emergent adverse event.

**Table S4.** Plasma concentration of erdafitinib.

| 8 mg (QD) | C_max_ (SD) [n] |
| --- | --- |
| Plasma concentration of erdafitinib (ng/mL)^a^ on C1D1  3h  6h  24h | 354 (178) [31]  340 (145) [31]  253 (132) [31] |
| Plasma concentration of erdafitinib (ng/mL)^a^ on C1D14^b^  Pre-dose  3h  6h  24h | 773 (394) [27]  1026 (461) [26]  1007 (446) [26]  803 (383) [26] |
| Plasma concentration of erdafitinib (ng/mL)^a^ on C2D1^c^  Pre-dose  3h  6h  24h | 633 (298) [9]  880 (301) [9]  855 (295) [9]  643 (245) [9] |
| Plasma concentration of erdafitinib (ng/mL)^a^ on C3D1^d^  Predose | 726 (435) [4] |
| Plasma concentration of erdafitinib (ng/mL)^a^ on C4D1^e^  Predose | 559 (203) [3] |
| 10 mg (7 day on/7 day off) | Erdafitinib |
| Plasma concentration of erdafitinib (ng/mL)^a^ on C1D1  3h  6h  24h | 355 (163) [4]  407 (131) [4]  337 (86.5) [4] |
| Plasma concentration of erdafitinib (ng/mL)^a^ on C1D7^f^  Pre-dose  3h  6h  24h | 1062 (158) [3]  1371 (391) [3]  1433 (140) [3]  1113 (187) [3] |
| Plasma concentration of erdafitinib (ng/mL)^a^ on C2D1^c^  Pre-dose  3h  6h  24h | 716 (869) [2]  1880 (-) [1]  1280 (-) [1]  1680 (-) [1] |

Abbreviations: C_max_, maximum serum concentration; C, cycle; D, day; h, hour; QD, once daily; SD, standard deviation.

^a^Plasma concentration data presented as mean (SD) [n] by nominal time. ^b^C1D14 data presented here only include those patients who received the same dosing schedule from C1D1 to C1D14 without any dose modification. ^c^C2D1 data presented here only include those patients who received the same dosing schedule from C1D1 to C2D1 without any dose modification. ^d^C3D1 data presented here only include those patients who received the same dosing schedule from C1D1 to C3D1 without any dose modification. ^e^C4D1 data presented here only include those patients who received the same dosing schedule from C1D1 to C4D1 without any dose modification. ^f^C1D7 data presented here only include those patients who received the same dosing scheduling from C1D1 to C1D7 without any dose modification.

**Supplementary Figure S1.** Patient flow.


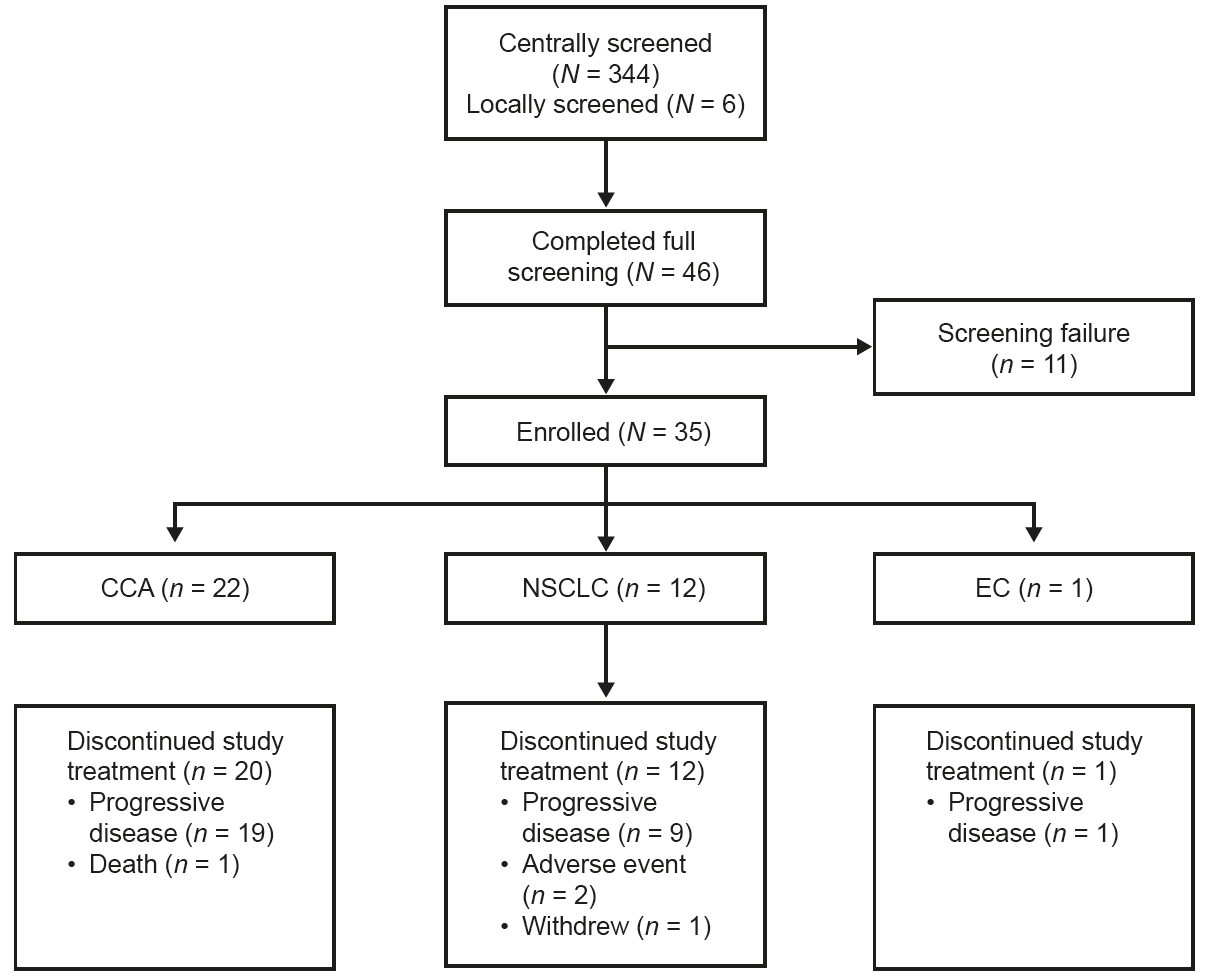


Abbreviations: CCA, cholangiocarcinoma; EC, esophageal cancer; NSCLC, non-small cell lung cancer.

**Supplementary Figure S2.** Maximal percentage reduction of sum of target lesion diameters from baseline for NSCLC.


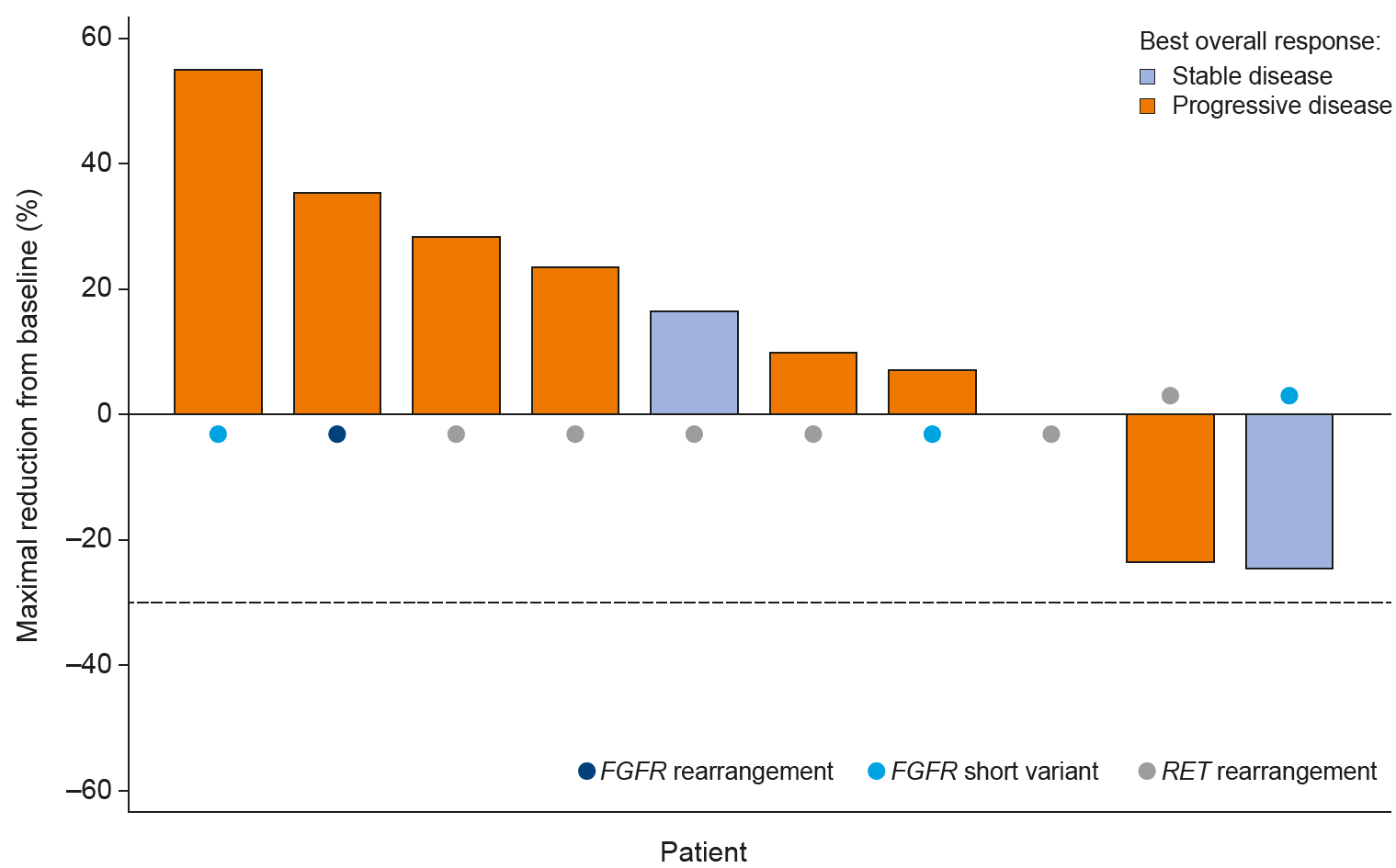


Note: Among the 12 treated patients with NSCLC, two were without post-baseline target lesion assessment, thus they were not reflected in this graph (one patient was not evaluable due to study withdrawal before the first scheduled post-baseline disease assessment and the other had progressive disease due to a new lesion discovered during an unscheduled visit before the first scheduled post-baseline disease evaluation). Three patients had stable disease. Among them, one had a maximal reduction from baseline of sum of target lesion of 0%.
